# Genetic structure of important resident brown trout breeding lines in Poland

**DOI:** 10.1007/s13353-020-00548-6

**Published:** 2020-02-10

**Authors:** Rafał Bernaś, Anna Wąs-Barcz

**Affiliations:** 1 Inland Fisheries Institute in Olsztyn, Department of Migratory Fishes, Rutki 49, 83-330 Żukowo, Poland; 2 National Marine Fisheries Research Institute, Department of Fisheries Resources, Kołłątaja 1, 81-332 Gdynia, Poland

**Keywords:** Brown trout, Hatchery lines, Stocking

## Abstract

The history of brown trout *Salmo trutta* L. stocking has long tradition in the European Union and other countries. Hundreds of hatchery facilities on continent have artificial broodstocks used for enhancement of neighbouring and also geographically far river basins. These practices have substantial effect on wild brown trout populations. To illuminate this phenomenon, eleven hatchery stocks and wild populations from northern Poland and Carpathian region were analysed using 13 microsatellite markers. Obtained results revealed high genetic diversity between studied stocks and clear differentiation between northern and southern populations and hybridization between these two major clads. As a recommendation, the principle of treating regions as metapopulations should be applied, which, in the case of Poland, means using the division of the northern and southern genetic lines that were revealed in the present study.

## Introduction

The brown trout, *Salmo trutta* L., is a European species with a range of occurrence that extends from the north of Iceland and northern Scandinavia and Russia to the south of the Atlas Mountains in north Africa, and from the Ural Mountains in the northeast and the Aral Sea basin in the southeast (Elliott 1994; Williams and Aladin 1991; Jonsson and Jonsson 2011). This species is polymorphic and several life strategies occur within it. The anadromous form of the species is referred to as migratory sea trout, and it undertakes migrations from its natal stream to the sea to feed, grow, and mature before returning to its birthplace to spawn. The resident form known as the brown trout follows another life strategy. It spends its entire life in fresh water and often spawns in the smaller tributaries of the stream it inhabits (Elliott 1994). Anadromous sea trout populations that migrate to the sea have a smaller range of occurrence that stretches to the north of the 42nd parallel in Western Europe in streams that drain into the White, North, Baltic, and Irish seas; the English Channel; and the Atlantic Ocean to the Bay of Biscay in northern Portugal. It does not occur in the Mediterranean drainage basin, but it does occur in the Black and Caspian seas (Elliot 1994; Klemetsen et al. 2003). Currently, approximately 400 migratory sea trout populations occur in the Baltic Sea (ICES 2019). Poland has approximately 25 sea trout rivers, which are those in which this species spawns naturally, and these are primarily in the Pomeranian region, but they are also located in the Vistula and Oder river drainage basins. Historically, the range of occurrence of this species was substantially larger with the largest spawning grounds located in the Carpathian tributaries of the Vistula River that were rendered inaccessible by the construction of numerous migration barriers (Bartel et al. 2007). The resident brown trout has a much larger range of occurrence that covers most of the historic range of the migratory sea trout and includes new locations of occurrence that have been created by stocking. The two forms of the species are important economically and are targets of both commercial and recreational fisheries. In the face of declining abundance and continually increasing anthropogenic pressure, methods are being sought to improve spawning and to rear fry. Stephan Ludwig Jacobi is considered to be one of the pioneers of salmonid artificial spawning as he developed a method for artificial spawning in the mid eighteenth century in Westphalia (Booke 1977). This method spread across Europe and developed slowly until the 1950s when a huge increase in hatchery production was observed. In Poland, stocking streams with salmonids began on a small scale in the second half of the nineteenth century in an attempt to compensate for overexploitation and deteriorating environmental conditions (Kołder 1958). However, mass stocking in numbers reaching hundreds of thousands of individuals did not begin until the end of the 1960s and 1970s (Dębowski 2018). Classically, analysis based on the mitochondrial DNA control region indicates five main evolutionary lineages of brown trout in Europe: Atlantic, Danubian, Mediterranean, Adriatic, and Marble. The Danubian lineage has spread from the Black Sea to the Caspian and Aral basins, the Atlantic lineage originally came from the rivers of the Atlantic basin, north Morocco, and Sicily, while the Mediterranean, Adriatic, and Marble lineages overlap with the Mediterranean basin (Bernatchez 2001). More detailed studies have revealed other subgroups like those in the Duero basin (Suárez et al. 2001), the Tigris basin (Bardakci et al. 2006), and the Balkan cluster (Marić et al. 2006). The genetic population structure of brown trout in Central Europe has been investigated in both wild and hatchery stocks in Austria (e.g. Weiss et al. 2001), the Czech Republic and Slovakia (e.g. Kohout et al. 2012), Germany (e.g. Lerceteau-Köhler et al. 2013), Hungary (Ősz et al. 2018), Poland (e.g. Wenne et al. 2016), Slovenia (e.g. Jug et al. 2005), and Switzerland (e.g. Keller et al. 2011). However, in Poland, only anadromous populations have been investigated in detail. Generally, in many cases, the results of these studies indicate that natural brown trout populations are affected by stocking with no native lines, and the present share of the Danubian and Atlantic haplotypes is hard to interpret. Furthermore, Lerceteau-Köhler et al. (2013) and Schenekar et al. (2014) suggest that the phenomenon of hybridization between the Atlantic and Danubian lineages could also have emerged through natural processes caused by multiple colonization processes in the post-glacial period. The history of mixing brown trout populations in Europe is a very long one, and many populations throughout Europe have been or are affected by this to some degree. Examples of mixed genetic lines can be also found in almost all European countries (e.g. Almodóvar et al. 2006; Apostolidis et al. 1996; Berrebi et al. 2019; Fruciano et al. 2014; Largiadèr and Scholl 1995). Numerous studies have addressed the fitness of salmonid hatchery fish in comparison with native stocks, and their performance has usually been determined as worse than that of original stocks. In many examples, non-local stocks have reduced survival rates compared with natural populations (Araki et al. 2008). Stocking with distant genetic lines can be harmful for native stocks and can result, inter alia, in outbreeding depression and lowered survival in subsequent generations (Ågren et al. 2019) or in the loss of local adaptations (Wang et al. 2002). Brown trout is a key angling species in many European rivers, and, consequently, this is associated with a significant degree of exploitation of this species and its supplementation through stocking practices. Additionally, stocking requirements encompassed in expert fisheries documents and the tendency to increase stocking quotas in tenders for fishery exploitation rights mean that implementing stocking plans using material produced locally is often impossible. This leads to the use of material from lines that are geographically and genetically distant. Bearing in mind these circumstances and the proper management of resources that preserves biodiversity, the main aim of the present research was to determine the genetic variation and mutual relations among different lines of hatchery reared forms of resident brown trout used for stocking in Poland and to compare them with selected wild populations, including anadromous ones. The hatchery stocks examined have never been studied and the results obtained are important not only from the perspective of basic studies but also as management recommendations for brown trout populations in Central Europe.

## Material and methods

### Sample collection

**Fig. 1:**
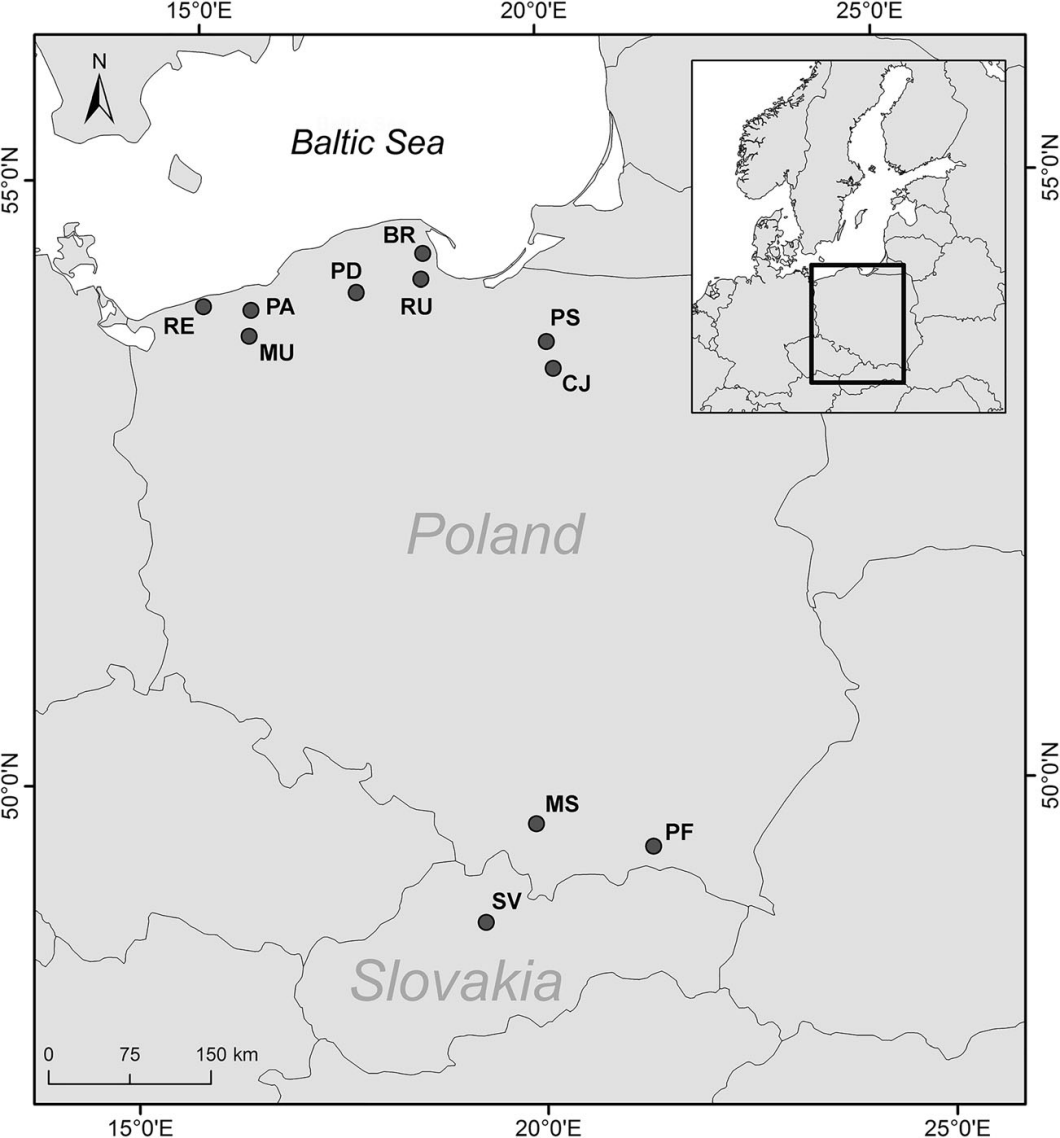
Map showing locations of the brown trout (*Salmo trutta*) stocks sampled and analysed

In total, 434 brown trout (*Salmo trutta* L.) from 11 lines of hatchery and wild populations were analysed; nine of these were resident lines and two were anadromous. Samples were collected so as to enable capturing the level of variation among the individual lines from the Carpathian region and from northern Poland. Detailed analysis of the samples is presented in Table 1 and Fig. 1. Sample abbreviations are as follows: PF, Folusz; SV, Slovryb; MS, Raba; PS, Pasłęka; CJ, Czarci Jar; BR, Rumia; PD, Dąbie; RU, Rutki; MU, Mogilica; PA, Parsęta; RE, Rega (Fig. 1). The hatchery in Folusz, founded in 1930, is one of the oldest in Poland (Kołder 1948). The current broodstock of resident brown trout at Foluszu (PF) was created about ten years ago using fish from the Leśnica, Hołcyna, and Węgierski Potok streams located in the upper Vistula drainage basin. Rearing was initiated with 655 individuals caught in these tributaries. Currently, the broodstock numbers about 700 females, and breeding is conducted in a closed cycle without supplementing the stock. The broodstock at the Slovryb (SV) hatchery, which is located in central Slovakia near Ružomberok on the Biely Potok stream, was created about ten years ago and is maintained in a closed cycle. In the past several years, stocking material from this facility has been released throughout Poland. The brown trout stock from the Raba River (MS) was created in 2001 in Myślenice with 145 individuals caught in several tributaries of the lower Raba River, mainly the Kobylok. The stock was supplemented once in 2006 with fish that also came from this tributary. Currently, artificial spawning is conducted using selections based on the quantitative trait of red dots on the dorsal fin, and the number of females used annually for breeding does not exceed 100 individuals. The Pasłęka River is 186.62 km long and its drainage basin has an area of 2294 km^2^. It drains into the Vistula Lagoon. Electrofishing is conducted annually in the Pasłęka River (PS) and its tributaries (mainly the Trojanka and Łukcianka) during the spawning season, and the spawn obtained is transported to the hatchery in Komorowo, where it is incubated and where the local spawning broodstock is maintained. Czarci Jar (CJ) is one of the oldest facilities in continuous operation producing stocking material in northern Poland. It was founded in the 1950s, and it has played a substantial role in stocking the rivers of northern Poland. Breeding is conducted in a closed cycle without supplementing the stock and number of functional females is about 300 specimens per year. The hatchery in Rumia was founded in the 1960s, based on local fish, and currently the broodstock in Rumia (BR) is maintained in a closed cycle. Dąbie (PD) is a modern hatchery and the closed broodstock was created in 2003 with fish from a facility in Kębłowo. Currently, the broodstock numbers approximately 3000 individuals. Rutki (RU) is the salmonid hatchery of the Inland Fisheries Institute (IFI) in Olsztyn, and it was founded in the late 1970s. The resident brown trout broodstock was created in the 1990s using individuals from the Radunia River and individuals from the hatchery in Łopuszna (southern Poland). The broodstock numbers approximately 200 females. The Parsęta (PA) is the largest of the Pomeranian rivers with a length of 157 km and a drainage basin of 3048 km^2^. The ichthyofauna of the river includes more than 20 species of fish and lampreys, but the dominant species are sea trout, *Salmo trutta* L., and the river bullhead, *Cottus gobio* L. (Dębowski 1999). It drains directly into the Baltic Sea. Samples were collected in the middle river segment from adult anadromous fish during spawning migration.

**Table 1:** Numbers of brown trout specimens examined, sampling date, place, age, ecological form, origin, and basin

Stock	*N*	Date	Place	Age	Form	Origin	Basin
PF	33	Fall 2017	Folusz	Parr	Resident	Hatchery	Vistula River, Baltic Sea
SV	44	Fall 2017	Slovryb	Parr	Resident	Hatchery	Danube, Black Sea
MS	46	Fall 2018	Myślenice	Adults	Resident	Hatchery	Vistula River, Baltic Sea
PS	35	Fall 2018	Pasłęka	Adults	Resident	Wild/hatchery	Baltic Sea
CJ	27	Spring 2017	Czarci Jar	Fry	Resident	Hatchery	Vistula River, Baltic Sea
BR	54	Spring 2017	Rumia	Fry	Resident	Hatchery	Baltic Sea
PD	40	Fall 2016	Dąbie	Adults	Resident	Hatchery	
RU	30	Fall 2016	Rutki	Adults	Resident	Hatchery	Radunia River, Baltic Sea
MU	37	Summer 2017	Mogilica	Parr	Resident	Wild	Parsęta River, Baltic sea
PA	44	Fall 2017	Parsęta	Adults	Anadromous	Wild	Baltic Sea
RE	44	Fall 2016	Rega	Adults	Anadromous	Wild	Baltic Sea

The Mogilica River (MU) is a left-bank tributary of the Parsęta River. It reaches a length of 44 km, and its basin covers an area of 150.43 km^2^. Individuals analysed in the present study originate from the upper segment of this stream, which is isolated by barriers constructed on it. This isolated population occupies a small area of about 65 km^2^ of the basin. The Rega (RE) is the longest of the Pomeranian rivers at 179 km, and its drainage basin has an area of 2700 km^2^. The predominant species of its ichthyofauna are *Salmo trutta* L. and *Cottus gobio* L. (Radtke et al. 2010). This river also drains directly into the Baltic Sea. Samples were collected in the lower river course from adult anadromous fish during spawning migration.

### DNA isolation and microsatellite amplification

Genomic DNA was extracted from fin tissue preserved in 96% ethanol using a Genomic Mini Kit (A&A Biotechnology) and diluted to a concentration of 30–100 ng. A set of 13 polymorphic and fluorescently labelled microsatellite loci (*OneU 9*, *Strutta 58P*, *Ssosl 438*, *Ssosl 311*, *Str15INRA*, *Str 543INRA*, *Str 60INRA*, *Str 73INRA*, *Ssos l417*, *Str 85INRA*, *Ssa 85*, *Bs131*, *Ssa 407*) was applied in a single multiplex PCR reaction using Qiagen Multiplex PCR Kit (Qiagen, Germany). The 7-μl multiplex PCR reaction was performed with about 100 ng of template DNA, 1× multiplex PCR master mix, and 0.2–0.6 μM of each primer. Amplifications were carried out in a TProfessional Basic Gradient thermal cycler (Biometra) with an initial heat of 95 °C for 5 min followed by 38 cycles of denaturation at 94 °C for 30 s, annealing at 55 °C for 90 s, and extension at 72 °C for 60 s. The PCR was terminated after 30 min and the final extension was at 60 °C. PCR products were genotyped in single capillary electrophoresis on an ABI Prism 3130xl genetic analyser (Applied Biosystems) along with GeneScan 600LIZ size standard (Applied Biosystems). DNA fragments were estimated using a Peak Scanner v1.0 (Applied Biosystems).

### Statistical treatment

Observed and expected heterozygosity was calculated using Arlequin 3.5.2.2. (Excoffier and Lischer 2010). Population-specific *F*_IS_ and pairwise-weighted *F*_ST_ values over all loci based on the number of different alleles were also determined with this software. Departures from the Hardy–Weinberg equilibrium were detected with chi-square tests in GenAlex 6.5 (Peakall and Smouse 2012). Overall, the F-statistic was estimated by analysing molecular variance (AMOVA) implemented in Arlequin 3.5.2.2 (Excoffier and Lischer 2010). HP-RARE was used to calculate allelic richness and the richness of private alleles (Kalinowski 2005). STRUCTURE 2.3.4 was used to detect genetic structure and gene flow (Pritchard et al. 2000). The Evanno method (∆*K*) was used (Evanno et al. 2005) to infer the true number of clusters (*K*) based on the rate of change in log probability among consecutive *K* values, which ranged from *K* = 1 to *K* = 12 with a burn-in and Markov chain Monte Carlo length of 10,000 each (200,000 burn-in and 500,000 Markov chain Monte Carlo replicates when analysing for hierarchical within-cluster structure) for five independent runs per *K* value. To this end, the Clumpak program was employed to identify the optimal alignment of inferred clusters across different values of *K* (Kopelman et al. 2015). Additionally, genetic heterogeneity was tested with the assignment test and the leave one out method in ONCOR. The algorithm records the fraction of assignments for each population that were correct and the population to which the individuals were most often incorrectly assigned (Kalinowski et al. 2007). Finally, the POPTREE2 program was used to create a neighbour-joining tree using *D*_A_ distances (Nei 1978) with 10,000 bootstrap replications (Takezaki et al. 2010).

## Results

**Fig. 2:**
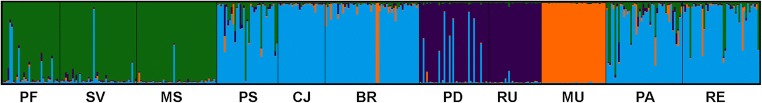
Clustering of 434 specimens from eleven stocks with putative *K* = 4. Each individual is represented by a column divided into *K* shades with each shade representing a cluster

**Fig. 3:**
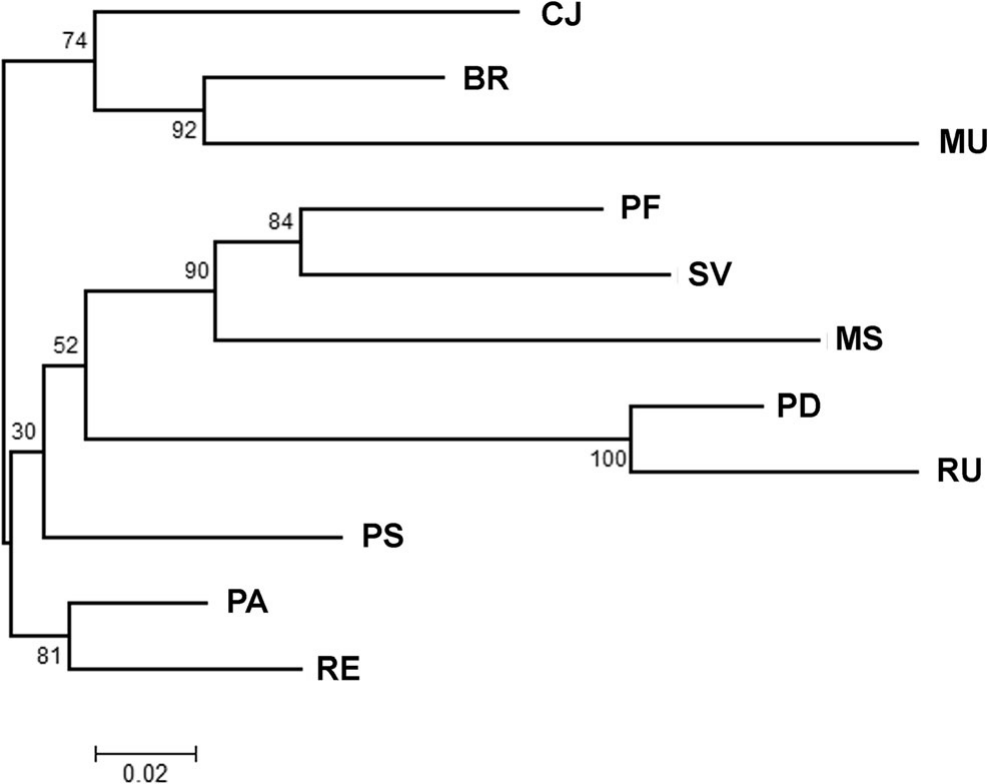
A neighbour-joining tree based on Nei’s distances among the eleven brown trout stocks. Bootstrap probabilities are shown on the tree

All 434 brown trout samples amplified successfully for all 13 loci. The mean number of alleles per population ranged from 3.62 (MU) to 10.39 (PA). Observed heterozygosity varied between 0.48 (MU) and 0.71 (PF, SV, and PS), and expected heterozygosity ranged from 0.50 (MU) to 0.74 (SV) per locus/population (Table 2). Eleven deviations from the Hardy–Weinberg equilibrium (*P* ≤ 0.01) were found after chi-square tests (Table 2). Allelic richness was greatest in the Folusz line (PF) at 9.01 and in the anadromous population from the main Parsęta River (PA) 8.91. By contrast, the lowest values were observed in the population from the Mogilica River (MU) and the closed hatchery lines Dąbie (PD) and Rutki (RU) at 3.54, 4.68, and 4.72 respectively (Table 2). The highest number of private alleles per population was estimated in the Folusz (PF, 0.85) and Slovakian (SV, 0.89) lines. Very low values of this parameter were found in the Raba River, Czarci Jar, and Dąbie hatcheries (MS, CJ, 0.03; PD, 0.05). Pairwise *F*_ST_ values were significant before and after Bonferroni correction (*P* ≤ 0.05) for all the tests (Table 3). The highest pairwise difference was indicated between the hatchery lines (RU) and (PD) and the Mogilica River (MU) *F*_ST_ = 0.24. By contrast, the lowest *F*_ST_ = 0.01 was found between anadromous populations from the Parsęta and Rega rivers (PA and RE) (Table 3). The lowest pairwise difference for resident lines was noted for the Folusz (PF) and Slovakian (SV) lines at 0.02. Overall, the *F*_ST_ obtained by AMOVA for all pairs of loci was 0.088 and significant; this can be interpreted as high genetic polymorphism among the studied groups. The highest percentage of variation was detected within individuals 89.9%. Overall *F*_IS_ and *F*_IT_ reached, respectively, 0.019 and 0.105 and were significant (*P* < 0.05). The Mogilica (MU), Rutki hatchery (RU), and the Raba stock (MS) samples showed the best assignment to the native groups based on the leave one out method test, with an accuracy of 100, 93.5, and 93.3% of correct fits (Table 4). The largest misidentification was for the anadromous lines from the Rega (RE) and Parsęta rivers (PA), both 25% (Table 4). Bayesian clustering methods were applied to examine genetic relationships among the eleven brown trout stocks and to provide information about the assignment of particular individuals to groups based on their genetic similarity. The results obtained using the Evanno method (Evanno et al. 2005) showed that the mean log likelihood against *K* plateaued at *K* = 4 with a maximum value of Δ*K* of *K* = 4 (Δ*K* = 562.04). At *K* = 4, the three lines from the Carpathian mountains (PF, MS, and SV) were clustered together. The next clade contained the line from the Pasłęka River (PS), northern hatcheries Czarci Jar (CJ) and Rumia (BR), and anadromous populations from the Rega and Parsęta rivers (RE and PA). The third clade was comprised populations from the upper Mogilica River (MU). The fourth clade consisted of breeding lines from the Dąbie (PD) and Rutki (RU) hatcheries (Fig. 2). The results of Bayesian clustering are in line with results obtained from phylogenetic analysis. A neighbour-joining (NJ) tree was built and the branches were supported by bootstrapped values. The NJ calculation showed that the genotypes examined belonged to four major clusters, which corresponded to Bayesian analysis (Fig. 3).

**Table 2:** Basic statistics of eleven brown trout stocks from the southern Baltic and the Carpathian area. *N*, number of analysed fish; *M*_*NA*_, mean allele number; *H*_*O*_, observed heterozygosity; *H*_*E*_, expected heterozygosity; *A*_*R*_, allelic richness; *P*_*AR*_, private alleles; *DHWE*, Hardy–Weinberg equilibrium deviations; *F*_*IS*_, stock-specific inbreeding coefficient (significant values are italicized)

Stock	*N*	*M*_NA_	*H*_O_	*H*_E_	*A*_R_	*P*_AR_	DHWE	*F*_IS_
PF	33	9.77	0.71	0.72	9.01	0.85	1	0.02
SV	44	10.23	0.71	0.74	8.85	0.89	2	*0.05*
MS	46	6.31	0.69	0.69	5.87	0.03	1	0.00
PS	35	9.15	0.71	0.72	8.41	0.45	1	0.01
CJ	27	5.77	0.62	0.61	5.69	0.03	0	− 0.01
BR	54	7.54	0.69	0.68	6.71	0.18	1	0.00
PD	40	4.92	0.54	0.54	4.68	0.05	0	0.00
RU	30	4.85	0.59	0.58	4.72	0.13	1	− 0.01
MU	37	3.62	0.48	0.50	3.54	0.1	3	*0.06*
PA	44	10.39	0.64	0.70	8.91	0.26	1	*0.08*
RE	44	10.00	0.71	0.70	8.67	0.36	0	− 0.01

**Table 3:** Genetic diversity indices for the eleven investigated brown trout stocks. *F*_ST_ values for pairwise comparisons of eleven brown trout stocks, which were all significant (*P* = 0.05), are below the diagonal; the average numbers of within-stocks pairwise differences are on the diagonal in italic characters; Nei’s genetic distances *D*_A_ are above the diagonal

	PF	SV	MS	PS	CJ	BR	PD	RU	MU	PA	RE
PF	*9.348*	0.272	0.494	0.368	0.761	0.548	0.995	1.036	1.702	0.420	0.534
SV	0.028	*9.673*	0.526	0.330	0.847	0.480	0.947	0.837	1.748	0.444	0.551
MS	0.051	0.053	*8.968*	0.606	1.059	0.695	1.263	1.186	1.721	0.485	0.655
PS	0.038	0.033	0.062	*9.376*	0.544	0.290	0.744	0.922	1.612	0.198	0.276
CJ	0.080	0.085	0.109	0.058	*7.962*	0.389	0.877	1.340	1.705	0.430	0.576
BR	0.057	0.049	0.072	0.031	0.043	*8.901*	0.820	0.971	1.505	0.292	0.462
PD	0.110	0.101	0.135	0.084	0.106	0.092	*7.011*	0.343	2.191	0.785	1.173
RU	0.109	0.086	0.123	0.097	0.147	0.103	0.045	*7.558*	2.225	0.838	1.193
MU	0.178	0.175	0.179	0.170	0.194	0.159	0.244	0.242	*6.512*	1.059	1.327
PA	0.044	0.045	0.051	0.021	0.047	0.031	0.088	0.090	0.115	*9.093*	0.143
RE	0.055	0.055	0.068	0.029	0.062	0.049	0.126	0.123	0.140	0.015	*9.131*

**Table 4:** Proportion of baseline individuals correctly assigned to their own stocks and share of largest misidentifications (%)

Stock	*N*	% correctly assigned	% of largest misidentification	
PF	32	84.40	9.40	SV
SV	44	90.90	6.80	PF
MS	46	93.50	4.30	PF
PS	35	65.70	14.30	RE
CJ	27	81.50	7.40	BR
BR	54	87.00	3.70	CJ
PD	40	82.50	17.50	RU
RU	30	93.30	6.70	PD
MU	44	100.0		
PA	44	59.10	25.00	RE
RE	44	52.30	25.00	PA

## Discussion

The lowest values of expected and observed heterozygosity in the populations studied were confirmed in the isolated population from the upper Mogilica (MU) and in the closed stocks from the Dąbie (PD) and Rutki (RU) hatcheries. At the two hatcheries, this was not the result of the small number of individuals used for spawning since the sizes of the broodstocks were considerable, and at Dąbie it was even very large. This could be associated with the small number of individuals in the founder stock (e.g. Verspoor 1988; Aho et al. 2006) or with genetic drift (e.g. Jorde and Ryman 1996; Campos et al. 2006). Because of sanitary requirements, these stocks are maintained without supplementation from wild populations. The small degree of variety in the group of individuals from the upper Mogilica (MU) is most likely the result of genetic isolation caused by the presence of impassible barriers in the stream (e.g. Marshall et al. 1992; Heggenes and Røed 2006). This is why the genetic distance between these and the other populations is the greatest. In addition to heterozygosity parameters, the allele richness values among specimens from these three groups were also the lowest. However, the presence of private alleles was the lowest in the groups of specimens from the Dąbie hatchery (PD) and the Raba River (MS). The lines bred at the Folusz (PF) and Slovryb (SV) hatcheries and the population from the Pasłęka River (PS) had the highest values of these parameters. An interesting issue with regard to the stock from Folusz was that, despite its broodstock being founded by individuals from isolated tributaries, it exhibited high genetic variability. This was most likely the effect of the considerable size of the founder stock and the application of an appropriate strategy for selecting parental pairs (Campton 2004). In turn, the population from the Pasłęka River, which was previously, but also more recently, stocked with material (including the anadromous form) originating from different parts of Poland, also exhibited substantial variation that resulted from often mixing populations that are geographically distant. This was particularly evident in the results of the Bayesian analysis of genetic structure and of the NJ tree, which located this population between lines from the Carpathian area and the anadromous populations from the Pomeranian rivers. The history of stocking brook trout stocking into the Pasłęka River is difficult to reconstruct, but releases of stocking material of trout, anadromous sea trout, and lake trout are documented (data from IFI Olsztyn). From the 1960s to the 1990s, stocking was performed mainly with material from the Czarci Jar hatchery, but also with that from neighbouring rivers, such as the Bauda, Wąska, and tributaries of the Drwęca River (Gizela and Poburzanka), and also with Pomeranian sea trout and lake trout (data from IFI Olsztyn). In the 1970s, trout from the hatchery in Rumia (Kostecki 2014) were released, while trout from a hatchery in Zawoja (Carpathian area) were released in 2000, and then later also material from the hatchery in Rumia was released (data from IFI Olsztyn). This complicated stocking history is reflected in the genetic structure. The fact is that repeated releases of fry from Drwęca tributaries (Gizela and Poburzanka) in which the anadromous form spawns and also releasing fry from other anadromous populations had an impact on the current genotype of trout in the Pasłęka River. What is important is that the presence of Carpathian genotypes is also clearly apparent. However, despite the releases over many years of trout material from the hatchery in Czarci Jar, the current line does not form a clear common group with that of the Pasłęka. The genetic distance between the fish from the hatchery in Rumia and the Pasłęka population is substantially smaller. Analysis performed with STRUCTURE indicated clearly that there are two hatchery genetic lines of the resident form in Poland: the southern (PF, MS) and the northern (CJ, BR). It is interesting that the genetic distance between the southern and the northern hatchery lines and the Pomeranian anadromous population was at a similar level. The least genetic diversity was observed between the anadromous populations from the Parsęta and Rega rivers, which was certainly the result of gene flow between them (low homing) (Dębowski and Bartel 1995) and earlier mixing of the sea trout populations from the Pomeranian regions. Such practices have not been in use since the 1990s, and the current anadromous Pomeranian population is gradually differentiating itself (Wenne et al. 2016). Genetic structure analysis also indicated that the hatchery lines from Rutki and Dąbie were, at some stage, of similar origin, but more genotypes occurred in the Dąbie stock that were similar to those of the Rumia or Czarci Jar stocks. The broodstock from Bialy Potok (SV) in Slovakia was previously studied using microsatellite DNA and mitochondrial markers. It was concluded that the Atlantic mtDNA haplotype dominated the line and that microsatellite clustering was grouped with populations from the Vistula drainage basin (Kohout et al. 2012). The results of the present research confirmed that this brown trout line was grouped with populations from the Vistula drainage basin and that the genetic distance between them and the southern Polish lines was small. That the Atlantic lineage dominated in this stock could be related to relocations and stocking, which Kohout et al. (2012) also suggest. However, as Weiss et al. (2001) demonstrate, the upper part of the Danube River basin is largely a habitat of the Atlantic lineage, and it cannot be ruled out that the occurrence of it in the (SV) line in some way also arises from natural process. Additionally, a high proportion of the Danube mtDNA phylogenetic group was found in populations from the upper Vistula River basin (Kohout et al. 2012), which suggests that there was some contact among populations from the Danube and Vistula basins in post-glacial times. On the other hand, the question arises as to the origin of differences among northern lines in Poland and populations from the upper Vistula river basin. Did these result from just a different post-glacial history or also from different factors such as homing? What is known from studies on extinct Atlantic salmon populations from the Oder and Vistula rivers is they were separated by relatively large genetic distance that probably resulted from recolonization from close but separate refugia that were deglaciated at different times (Bernaś et al. 2016). This was probably similar in the case of brown trout, but the difference was that low homing in anadromous brown trout populations reduced the genetic variability of neighbouring populations (Degerman et al. 2012). Analysis of mtDNA haplotypes in Bernatchez (2001) shows the existence of different haplotypes in anadromous populations from the Pomerania region and from the Vistula River, while it also indicates a lack of other, e.g. Danubian, haplotypes in these populations. This might suggest that if any connection between the Danube and Vistula brown trout occurred, it originated from a northerly direction. Many studies indicate that introgression among native and non-local fish can lead to the loss of local adaptations by depleting original genetic variability, and it poses a risk of outbreeding depression in native stocks (e.g. Gilk et al. 2004; Machordom et al. 1999; Marzano et al. 2003). The results obtained in this study of brown trout populations from the Pasłęka River (PS) are good examples of the introgression effect where more than 50% of the fish analysed were dominated by the non-local genome. This is why applying the principle of stocking with local lines, especially those that are based on individuals that are caught in the wild, is a rational action to undertake even if there are no official regulations requiring this be done. The principle of treating regions as metapopulations should be applied, which, in the case of Poland, means using the division of the northern and southern genetic lines that were revealed in the present study.
